# Dr. E. L. Trudeau

**Published:** 1915-12

**Authors:** 


**Dr. E. L. Trudeau,** P. & S. 1871, the well known authority on tuberculosis, died at Saranac Lake November 15, aged 67.

